# Pediatric and Adolescent Oncofertility in Male Patients—From Alpha to Omega

**DOI:** 10.3390/genes12050701

**Published:** 2021-05-08

**Authors:** Ovidiu Bîcă, Ioan Sârbu, Carmen Iulia Ciongradi

**Affiliations:** 1 2nd Department of Morphofunctional Sciences—Cell and Molecular Biology, “Grigore T. Popa” University of Medicine and Pharmacy, 700115 Iași, Romania; ovidiubica.d@gmail.com; 2 2nd Department of Surgery—Pediatric Surgery and Orthopedics, “Grigore T. Popa” University of Medicine and Pharmacy, 700115 Iași, Romania; iuliaciongradi@yahoo.com; 3 Department of Pediatric and Orthopaedic Surgery, “Sfânta Maria” Emergency Children Hospital, 700309 Iași, Romania

**Keywords:** fertility preservation, prepubertal boys, cancer, oncofertility, pediatric, in vitro spermatogenesis

## Abstract

This article reviews the latest information about preserving reproductive potential that can offer enhanced prospects for future conception in the pediatric male population with cancer, whose fertility is threatened because of the gonadotoxic effects of chemotherapy and radiation. An estimated 400,000 children and adolescents aged 0–19 years will be diagnosed with cancer each year. Fertility is compromised in one-third of adult male survivors of childhood cancer. We present the latest approaches and techniques for fertility preservation, starting with fertility preservation counselling, a clinical practice guideline used around the world and finishing with recent advances in basic science and translational research. Improving strategies for the maturation of germ cells in vitro combined with new molecular techniques for gene editing could be the next scientific keystone to eradicate genetic diseases such as cancer related mutations in the offspring of cancer survivors.

## 1. Introduction

An estimated 400,000 children and adolescents aged 0–19 years will be diagnosed with cancer each year [1], the most common types being leukemias and lymphomas, central nervous system tumors, soft tissue sarcomas, neuroblastomas and kidney tumors. Due to continued advancement in medical therapies and improvements in the early detection of malignancy in patients who receive a cancer diagnosis, the five year survival rate has dramatically improved, and now is between 80–84% compared to the late 1970s when it was almost 60% [2,3,4]. The remarkable improvement in survivorship has prompted increased awareness of long-term quality of life concerns, including fertility impairment and premature testicular insufficiency among adult male survivors of childhood cancer. These issues became important to patients and their families, with future fertility being reported as a key concern [5,6].

## 2. Testicular Function—Fertility Reservoir

Testicular function is based on an intact hypothalamic-pituitary-gonadal (HPG) axis. The HPG axis drives male sexual development and fertility. This process is under the control of gonadotropin-releasing hormone (GnRH) which is produced in neurons scattered throughout the anterior hypothalamus in a pulsatile manner. GnRH in turn stimulates the synthesis and secretion of luteinising hormone (LH) and follicle stimulating hormone (FSH) from the anterior pituitary gland. LH and FSH are released into circulation in bursts and activate receptors on Leydig cells and Sertoli cells, respectively, which stimulate testosterone production and spermatogenesis [7,8].

Testicular function is composed of reproductive and androgenic function, and males may have a congenital (primary) or acquired (secondary) defect in reproductive function (spermatogenesis), androgenic function (testosterone) or both. In the assessment of male reproductive and androgenic testicular function, it is important to distinguish between primary and secondary causes [9].

## 3. Influence of Cancer Treatment on Fertility

A cancer diagnosis by itself is a risk factor for infertility, even before initiating treatment with surgery, radiation and/or chemotherapy. Gonadal damage is a common consequence of treatment for pediatric malignancies that leads to a high incidence of infertility that varies widely with age and gender [10,11].

Gametogenesis and hormone production are differentially sensitive to cancer diagnosis and treatment exposure in males. Certain types of cancers have an increased risk of azoospermia or diminished semen parameters even before starting any kind of treatment. In the literature, some studies demonstrated that leukemia and lymphoma are risk factors for pre-treatment azoospermia [12]. Results from a program made by Ragni et al. [13] reported that more than 10% of cancer patients who banked sperm at their institution were azoospermic before treatment. There are several studies that examined semen parameters in male patients with testicular cancer and reported that they have decreased sperm concentrations and parameters compared to male patients with different oncologic diagnoses [14].

In the male population, younger patients seem to be at higher risk than older patients. The prepubertal testis is considered very sensitive and vulnerable to radiation and cytotoxic chemotherapy because of the constant turnover of its undifferentiated spermatogonia. In the post pubertal testis, rapidly differentiating spermatogonia can be depleted by low dose chemotherapy or radiation. This results in a depletion of the remaining later stage differentiating cells and eventual oligospermia (<15 million sperm/mL of semen) or azoospermia (absence of sperm in the semen) which causes depletion of the testicular reserve or ”burnout” and premature testicular failure [15,16]. After damage due to chemotherapy or irradiation, the surviving spermatogonia stem cells turn into mitotically active spermatogonia, and along with the supporting Sertoli cells they become the foundation of spermatogenesis regeneration. When testicular damage is severe due to a high-dose cytotoxic environment, apoptosis is triggered in all subpopulations of spermatogonia cells and this leads to permanent sterility because they cannot support spermatogenesis.

It is important to highlight that impaired fertility can have a strong impact on patients’ quality of life by affecting sexual wellbeing, identity and self-esteem [6]. Due to assessment of the risk for impaired fertility after therapy, international guidelines including the American Society of Clinical Oncology (ASCO) and the National Institute for Health and Care Excellence (NICE), recommend the importance of integrating discussions about fertility into the provider–patient dialogue and to provide options for fertility preservation procedures before initiating any gonadotoxic therapy [17,18,19]. It has been cited that parents and adult male survivors of childhood cancer have a significant amount of regret because fertility preservation procedures were not discussed before starting any kind of treatment and over 75% of childhood cancer survivors expressed the desire to have children in the future [20].

### 3.1. Chemotherapy

Since the first treatments of chemotherapy in the post-World War II era, there have been many discoveries toward the development of more tolerable and targeted cancer treatments [21]. There are different classes of chemotherapeutic agents that can impair spermatogenesis. The first person to describe this phenomena was Spitz in 1948, when he reported that 27 of 30 men who had been treated with nitrogen mustard were azoospermic [22]. The effect of chemotherapy on the pre- and post-pubertal testis, and hence future fertility, varies and is mainly agent and dose dependent [12]. A history of chemotherapy is also often associated with increased post treatment gonadotropin (LH and FSH) levels, a sign that the pituitary gland is actively compensating the impaired testicular production of testosterone and sperm, respectively [23]. Dose-dependent treatments are seen within the sensitive spermatogonia stem cells, and impaired sperm production can last months to years prior to resolution. With prolonged or particularly hazardous treatment regimens, azoospermia can be permanent [24].

ASCO classifies the likelihood of infertility based on various chemotherapeutic regimens into low (<20%), intermediate (20–80%) and high (>80%) risk of infertility [25].

Broadly, it has been well documented that alkylating agents are toxic to testis in a dose-dependent fashion, and in patients receiving these agents, calculation of the right equivalent dose may help to quantify the risk of future infertility [26]. Alkylating chemotherapeutic agents work by disrupting DNA function via DNA base pair alkylation, formation of abnormal DNA cross-bridges and mispairing of nucleotides. These agents also impede DNA replication [27]. Normal sperm count typically recovers by 12 weeks post-therapy in patients treated with non-alkylating agents [28]. The risk of azoospermia is approximately 10% when the cyclophosphamide equivalent dose is less than 4 g/m^2^, whereas approximately one-quarter of individuals who receive more than this dose will retain a normal sperm concentration [28]. Spermatogenesis recovery is believed to be unlikely with doses of 19 g/m². The cyclophosphamide equivalent dose scoring system, available as an online calculator, can be used to compare gonadal toxicity of different alkylating agents [26].

Heavy metal (platinum-based, e.g., cisplatin and carboplatin) treatments damage DNA and interfere with DNA replication and can result in either a temporary or permanent suppression of spermatogenesis. There are several cisplatin-based regimens that temporarily impair spermatogenesis with a good recovery rate in a significant number of patients [27]. Antimetabolite therapy such as methotrexate and mercaptopurine appear to have no effect on male fertility. Chemotherapy protocols, such as MOPP, COPP or ABVD have shown high rates of long-term azoospermia. The potential gonadotoxic impact is associated with the fractionation schedule of the treatment and is clearly dose-dependent in cyclophosphamide-based regimens [29,30].

### 3.2. Radiotherapy

Radiation therapy for cancer has been used for more than 80 years and has implications in testicular function by potentially damaging both germ cells and Leydig cells, immature stem cells and spermatogonia, the latter being the most sensitive. Testicular tissue is extremely radiosensitive and even lower doses of direct radiation can impair spermatogenesis, more so than hormone production. However, both spermatogenesis and hormone production can be impaired after higher doses of radiation therapy are delivered to the hypothalamic/pituitary area.

Radiation to the testes markedly reduces the number of spermatocytes 2–3 weeks post-therapy with declines in ejaculated sperm count in the next 10 weeks. Azoospermia is typically present at 18 weeks post-therapy [16,31]. The extent of DNA damage to testicular germ cells and somatic cells is field, dose and fractionation dependent, and the testicle may be exposed directly (testicle is the intended target) or indirectly (result of a scatter when treatment is delivered to other organs/structures) [32].

Spermatogenesis is highly sensitive to radiation and doses as low as <0.1 Gy have been reported to affect testicular germ cell and cause oligospermia. Reversible short-term azoospermia has been identified at the dose of 0.35 Gy. Transient effect on spermatogenesis is common at very low doses, <2 Gy [32], whereas doses of 2–3 Gy may cause a long-term effect with the potential for recovery. Doses of more than 6 Gy can cause total depletion of spermatogonia stem cells and permanent sterility [33,34]. Pubertal development and sexual function are impaired with irradiation doses of at least 12 Gy. Primary hypogonadism may appear at radiation doses of 20–30 Gy to the hypothalamus pituitary axis, managed with androgen replacement [35,36]. Considering it takes almost 2 months to produce mature sperm, the effects associated with radiation exposure are typically delayed. These changes tend to first manifest 2 to 3 months after exposure [32].

### 3.3. Surgery

Some surgeries in male adolescent cancer can result in long-term negative influences for fertility potential. Testicular surgery can affect production of sperm and hormones or interfere with the transport of sperm [37].

Patients who underwent a retroperitoneal lymph node dissection (RPLND) or prostatectomy as part of the oncologic treatment plan can have a damaged autonomic nervous system. This is because during this intervention the sympathetic chain, which is neurologically responsible for driving ejaculation, may be transiently or permanently damaged. This impairment in sympathetic stimulation can manifest clinically as failure of seminal emission, anejaculation or retrograde ejaculation [33].

Other types of deep pelvic surgery can injure the parasympathetic and sympathetic nerves responsible for erection and ejaculation (via injuring the vas deferens) and place the patient at risk of erectile dysfunction or obstructive azoospermia [34].

### 3.4. Bone Marrow Transplantation

Bone marrow transplantation, which requires exposure to high-dose chemotherapy and/or total body irradiation, poses especially high risk to infertility and delayed puberty [38,39]. Conditioning regimens for bone marrow transplantation involving total body irradiation (10 or 13 Gy) have been reported to be highly associated with spermatogenesis failure, with an azoospermia rate in post-pubertal boys of 48–85% [40]. Incomplete pubertal development or pubertal failure have been reported to occur in approximately 53% of prepubescent males exposed to hematopoietic cell transplantation [39].

## 4. Fertility Preservation—Promising Times

Fertility compromise occurs in one-third of adult male survivors of childhood cancer [41]. Fertility preservation (FP) is an umbrella term for the range of medical and surgical interventions intended to minimize the adverse effects of cancer and its treatment on future fertility [42]. FP is a complex field with multiple considerations and offers treatments aimed at protecting future reproductive ability for individuals. Fertility preservation (FP) is the process of harvesting and storing gametes with the intent of offering an opportunity for biologically related offspring in the future. FP has been cited as one of the top five unmet needs for adolescent cancer patients, along with health, work/school, romantic relationships and close friends [42,43]. The American Society of Clinical Oncology (ASCO) in 2006 first published guidelines recommending that referral for FP be offered to patients of reproductive age regardless of gender, age or diagnosis. They state that, “As part of education and informed consent before cancer therapy, oncologists should address the possibility of infertility with patients treated during their reproductive years and be prepared to discuss possible fertility preservation options or refer appropriate and interested patients to reproductive specialists” [25].

As Taylor et al. stated, ”Fertility is a long-term issue influenced by short-term decisions,” in childhood cancer [44], increasing the relevance of quality-of-life issues such as fertility preservation which is an emerging crucial survivorship issue [45].

### 4.1. From Alpha—FP Counseling

There are several international societies all over the world that recommend the discussion of fertility risks and preservation options before commencing cancer treatment as part of routine care [17,18]. Patients and their families need information at diagnosis regarding the potential impact of therapy on fertility and decisions to pursue or forego a preservation procedure. Time means life in oncologic patients, and there is never a good time to discuss the risk of infertility with them. However, this discussion needs to occur at the right time, before the impairment of fertility has already happened, while taking into account the urgency of treatment initiation. Physicians play an important role in having this discussion and they need to balance the provision of fertility care with the need for timely diagnosis, disease staging and starting treatment. Every hospital should have a fertility preservation counsellor to start the discussion with a basic explanation of puberty, reproductive health, pregnancy and hormone regulation, as well as the impact of treatment and their FP options. Information provided to the patient should be developmentally appropriate and determined by the patient’s age, cognitive ability to grasp the topic and maturity level.

Research in the literature has reported on the desire of patients and their families to discuss FP counselling before or after treatment. Patients who were not informed about the impairment of fertility and their options had more negative moods and higher distress with objective measures [46,47]. Rotker et al. reported in 2017 in a retrospective chart review performed for men newly diagnosed with cancer that receiving even brief formalized nursing counselling prior to initiation of chemotherapy correlated with increased rates of sperm banking among cancer patients. This study included a total of 766 male patients and the rate of sperm banking for those patients who did receive counselling was significantly higher (17.6%) and the odds of banking increased 2.9 times for those who received counselling compared to those who did not. These results support the use of formalized fertility counselling for patients prior to initiation of chemotherapy [48].

As Runco said, “To maximize informed consent, a consultation with a specialist providing the fertility preservation procedures should be provided much as we have a surgeon to discuss surgical interventions and a radiation oncologist to discuss radiation.” An FP consult should be an integral part of the treatment plan and presented in the context of overall prognosis [49].

### 4.2. Oncofertility—A Bridge between Science and Biotechnology

The concept of oncofertility has been established in the medical community which brings together oncology with reproductive science formed by a multidisciplinary team including oncologists, hematologists, physicians, fertility specialists, scientists, counsellors, laboratory experts, ethicists, nurses and researchers all focused on maximizing fertility opportunities for patients.

With intensified interest in FP in cancer patients, the term oncofertility was coined and has since become its own area of clinical practice and research and should be an integral part of cancer care diagnosis through to survivorship [42,50].

### 4.3. Fertility Preservation—Beyond Oncology

Pediatric malignancies are not the only diseases treated with gonadotoxic agents. Other medical conditions that do not involve malignancy may also be an indication of fertility preservation. Organ-specific involvement and chemotherapy exposure in diseases such as juvenile systemic lupus erythematosus [51] and renal diseases, such as nephrotic syndrome [52] are examples that may use Cyclophosphamide in their treatment management and some of them may receive cumulative doses which can negatively impact gonadal function and reproductive outcomes. Current studies have an interest in FP outcomes of transgender youth and children with disorders of sexual development (DSD) [53]. Transgender medicine is an expanding field, and these adolescent and young adult patients are faced with fertility preservation challenges as they pursue hormone therapy for transition. Recent data suggest that utilization of fertility preservation procedures in transgender youth is low, and further research is needed to understand decision making influences in this population [54]. Children with DSD, where conditions such as Klinefelter chromosomal, gonadal or phenotypic sex is atypical, also have unique needs with regard to fertility preservation. Androgen insufficiency syndromes (as well as other XY DSD), congenital adrenal hyperplasia and mixed gonadal dysgenesis are associated with early gonadal failure, and in some cases, the risk of developing gonadal malignancy [55].

## 5. Fertility Preservation in Pubertal or Adolescent Boys

### 5.1. Sperm Cryopreservation

Cryopreservation consists of using very low temperatures to preserve structurally intact living cells and tissues [56]. The first successful pregnancy from frozen/thawed sperm in the setting of insemination was reported by Polge et al. in 1953, when they used human sperm cryopreservation [57]. Storing human sperm in cryoprotectants such as liquid nitrogen was an extremely important discovery and lead to further advancements. Cryoprotectant with low toxicity is needed because poor semen quality may have more damage, resulting in decreased ability for fertilization compared with normal samples [58].

The stage of pubertal development is considered the best indicator of spermarche (initiation of sperm production), with sperm cryopreservation typically offered to adolescents who are at least Tanner stages II to III for genital development, with motile spermatozoa reported and with a testicular volume of 10–12 mL sample [59,60,61]. For men and post pubertal adolescents who choose FP, masturbation with sperm cryopreservation remains the gold standard for management. It is non-invasive, relatively inexpensive and it has proven successful for long-term cryopreservation, with a report of one healthy live birth using sperm samples cryo-stored for 40 years [62]. Other studies report that live birth has been achieved with sperm that were cryopreserved for as long as 28 years [63].

When obtaining specimens via masturbation, spermatotoxic lubricants should be avoided and patients should be offered adequate privacy and time to ensure that the setting is optimized for specimen collection [64]. This approach can be accomplished at a reproductive medicine facility, at home with the specimen brought immediately to a reproductive medicine facility, or in a hospital. Some younger patients may not have attempted self-stimulation or masturbation before their cancer diagnosis, which can create a very difficult conversation for the physician, parents and patient.

### 5.2. Testicular Sperm Extraction (TESE)

Some patients, other than adolescent boys around 12–13 years old who are unable to produce semen through masturbation, may not ejaculate due to a multitude of factors including social, religious, cultural or medical (anxiety, erectile dysfunction, hypogonadism, pain, neurological impairment and medication side effects) [65]. Treatment with phosphodiesterase type-5 (PDE-5) inhibitors may facilitate an adequate sample, even in men without a history of erectile dysfunction [66]. For patients who did not preserve a semen sample and have persistent azoospermia after cancer therapy, there are several options to retrieve rare sperm directly from the testis during a surgical procedure called testicular sperm extraction (TESE). Some spermatogonia stem cells may survive the gonadotoxic therapy and produce focal areas of spermatogenesis in the seminiferous tubules. Picton et al. related results from a total of five centers and reported an overall sperm recovery rate of 44 % in azoospermic patients undergoing TESE after chemotherapy [60].

Alternatives to the procurement of semen samples by masturbation include methods such as:

- Penile vibratory stimulation (PVS): this is typically tried first and it is considered to be a non-invasive technique, as it can be used in the privacy of a patient’s home and does not require general anesthesia. These devices are usually quite affordable and typically have settings for variable amplitude and frequency. The vibratory pad of the device is placed on the ventral part of the penis near the frenulum, helping to trigger seminal emission and the ejaculation reflex [67].
- Electroejaculation (EEJ): a more invasive option, normally involving general anesthesia. This requires coordination with operating room and laboratory staff. It is more often used in patients who have failed PVS and is considered next-line therapy [68]. It can often be performed in the same anesthetic setting as additionally required oncologic procedures with minimal morbidity [69]. Hovav [70] reported that electroejaculation in six 15 to 18-year-old boys resulted in the patients successfully obtaining sperm in all cases. A study of 30 adolescents treated with electro-ejaculation demonstrated a sperm recovery rate of 60% [71].
- Surgical sperm extraction (from the epididymis or the testis): this is used when patients are unable to produce a specimen or present with azoospermia. This can be performed concurrently with other procedures such as central line placement, orchiectomy and bone marrow biopsy. Sperm can be potentially retrieved by percutaneous epididymal sperm aspiration, testicular sperm aspiration and micro-epididymal sperm aspiration [72].
- Micro-TESE: the use of an operating microscope with microsurgical testicular sperm extraction (micro-TESE) can assist in the identification of focal areas with active spermatogenesis. With this procedure, the risks of testicular damage (such as scrotal hematoma and skin discoloration, infection, persistent pain, and swelling) are minimal. For these reasons, TESE has become an emerging option for azoospermic patients with cancer and has been termed ”onco-TESE” [73,74].

The American Society of Clinical Oncology guidelines state that all males who have progressed into puberty and are able to provide a semen sample prior to initiation of therapy should be offered sperm cryopreservation as the only established mechanism of fertility preservation [17]. After cryopreservation, stored sperm can be thawed at a later date to achieve pregnancy by intrauterine insemination or in vitro fertilization (IVF) (with intracytoplasmic sperm injection (ICSI) if counts are low) [75,76].

### 5.3. Gonadal Shielding

Gonadal shielding can be used to protect the testes from scatter radiation using lead shielding. The proper shielding technique should be carefully evaluated on a case-by-case basis depending on total radiation dose, fractionation and the specific mode of delivery of the external beam therapy [77,78]. However, when the testicular tissue requires radiation therapy as a part of cancer treatment, shielding cannot be used. At other times, the proximity of the testes to the target of radiation results in scatter radiation to the testes which can also result in impaired spermatogenesis [79].

### 5.4. Testicular Tissue Cryopreservation (TTC) and Experimental Procedures: A Great Challenge for Prepubertal Male Patients

Currently, there are no proven fertility preservation techniques with cryopreservation of sperm for prepubertal patients, but there are several experimental strategies that might be used to restore spermatogenesis or fertility from cryopreservation of SSCs and testicular tissue [80,81]. Testicular tissue in prepubertal boys involves surgical removal of immature testicular tissue (an excisional biopsy through a trans-scrotal approach is ideally coordinated with another surgical procedure (e.g., biopsy and port placement) to minimize anesthetic risk and expedite initiation of treatment) prior to treatment, and cryopreservation via slow freezing [82]. Protocols are being investigated to enable intratesticular grafting of tissue or infusion of testicular cell suspension into the seminiferous tubules [82,83]. Eligibility for TTC generally includes prepubertal children with high risk of infertility or patients who are unable to provide an adequate semen specimen.

According to a recent survey from the Oncofertility Consortium at Northwestern University [84], there are currently at least 16 health centers around the world, seven of them in Europe [60], offering cryopreservation of testicular biopsies containing SSCs to prepubertal oncological patients. Most centers perform slow freezing, although vitrification has demonstrated to be effective in preserving human spermatogonia as well [85,86].

TTC is contingent on the future development of techniques for the maturation of spermatogonia stem cells (SSC) into sperm. In addition, cryopreservation of testicular tissue will maintain the microenvironment niche of the SSCs (mainly the somatic cells, Sertoli, Leydig and peritubular cells) which may increase the viability and functionality of the SSCs following tissue thawing when compared to thawed cryopreserved cells.

Systematic studies on prepubertal human testicular tissues with evaluation of both cell-based and tissue-based endpoints are needed. It is possible that the optimal freezing condition depends on the intended use of the tissue or cells. Cryopreservation of testicular tissue is still an experimental procedure that should be offered only to patients with a high risk of acquired azoospermia unable to produce sperm at the time of diagnosis [60]. A variety of SSC-based therapies have been previously described, which are discussed in detail below.

#### 5.4.1. Spermatogonia Stem Cell Transplantation

Among the strategies designed to restore spermatogenesis in cancer survivors using immature cryopreserved testicular tissue, SSC transplantation is probably the most promising approach [87] Male sperm development is a continuous process, and it starts at the time of puberty. SSCs present in the basement membrane of the seminiferous tubules of the testes can either terminally differentiate into spermatocytes or self-replicate, replenishing the germ cell pool. Spermatocytes undergo meiosis to form spermatids, which then undergo spermatogenesis and are poised to initiate sperm production. This plays a key role in consideration for fertility preservation as SSCs provide the basis for fertility preservation in prepubertal boys [88]. This complex self-renewal and differentiation process is under regulation by intrinsic factors including Bcl6b, promyelocytic leukemia zinc finger (PLZF), Octamer-4 (OCT4), zinc finger, broad complex/Tramtrack/bric-a-brac (ZBTB), the receptor KIT, proto-oncogene receptor tyrosine kinase (c-Kit) and neurogenin 3 (Ngn3). Extrinsic factors are also involved which include glial cell line-derived neurotrophic factor (GDNF), Fibroblast growth factor 2 (FGF2), colony stimulating factor 1 (CSF1) and WNT family member 5A (WNT5A) [89]. Multiple markers together must be used to confirm their isolation. Other SSCs surface markers include integrins a6 and b1, cadherin 1 (CDH1), GFRa1, ID4, ret protooncogene (RET), thymus cell antigen 1 (Thy-1) and a cluster of differentiation 24 (CD24). Major histocompatibility complex class I (MHC-I) and CD34 are not involved but can be stimulated by RA (Stra8) expression amongst others. This helps to differentiate SSCs from the surrounding testicular cells. Future research on identifying unique markers would simplify this process [90]. This self-renewal and differentiation capacity allow the organism to produce sperm if there are functioning SSCs. Methods to encourage SSC function outside of the normal host are being researched. SSCs transplantation was first described in 1994 when scientists demonstrated that SSCs could be isolated and transplanted to regenerate spermatogenesis in infertile recipient mice [91,92]. SSC transplantation has been successful in animal models, but this approach is still at the experimental stage for humans. It was reported that cryopreserved testicular cells from non-Hodgkin’s lymphoma patients were transplanted into their testes after recovery from cancer [91,92]; however, follow-up for these patients was not published. The main limitation of this technology in humans is the possible contamination of testicular cells with cancer cells that may reintroduce malignancy to the patient after recovery [93,94].

#### 5.4.2. De Novo Testicular Morphogenesis with the Introduction of SSC and Supporting Testicular Cells into a Decellularized Testicular Scaffold 

There are several studies in extension about testicular cells and their remarkable ability to reorganize to form normal looking seminiferous tubules when grafted under the skin of a recipient animal [95]. Kita et al. mixed fetal or neonatal testis cells from mice or rats with GFP+-cultured mouse germline stem cells and growth factor-reduced Matrigel and grafted under the skin of immune-deficient mice [96]. Seven to ten weeks after grafting, seminiferous tubules with complete spermatogenesis originating from both intrinsic germ cells and cultured (GFP+) germ cells were observed. Tubules were dissected and GFP+ round spermatids were recovered and injected into mouse oocytes. The resulting embryos were transferred to recipient females and gave rise to ten mouse pups, including four with the GFP transgene. The human experiment may be complicated by limited availability of fetal, neonatal or prepubertal human testis cells [97].

#### 5.4.3. Autologous Grafting and Xenografting of Testicular Tissue

Another alternative approach to restore spermatogenesis in cancer survivors is autologous immature testicular tissue grafting. This technique was originally described in a report where the engraftment of small pieces of prepubertal testicular tissue under the skin of immunosuppressed castrated host mice resulted in the production of sperm that could be retrieved for downstream Assisted Reproductive Technologies (ART) application [98]. This technique has given rise to sperm [99] and even healthy offspring in other species, including nonhuman primates [100]. When compared to testicular tissue xenografting, this technique may allow for more exposure of testicular cells to the new environment. Structural organization of the seminiferous tubules might influence the development of xenograft testicular tissue [101,102]. The promising results obtained from animal models, together with >15 years of cryopreserving immature testicular biopsies from pre-pubertal patients, encourages further research to restore fertility in azoospermic men [103,104]. Therefore, additional experimentation is merited. Testicular tissue grafting will not restore natural fertility but could generate haploid sperm that can be used to fertilize oocytes by ICSI.

Xenotransplantation, where cells from one species are transplanted into the microenvironment of another species, remains a useful tool in the growth of SSCs. The first documented occurrence of SSC xenografting from humans occurred in 2002, when testis biopsies of six infertile men were injected into the rete testis of nude mice. These were found to survive for up to 6 months in the host testes but with severely decreased numbers and without assaying their function [105]. This method has been performed with human samples. During the diagnosis of maturation arrest in humans, 16 human donor samples from eight patients were cultured in vitro, then transplanted into rete testis of immune deficient mice who had been rendered infertile by busulfan treatment. These cells proliferated in the host along the basement membrane, although no sperm were isolated. This suggests that complete differentiation may require human signaling factors [106]. There have been limited results in the xenografting of adult testis tissue. Although germ cells can survive in xenografting, no complete spermatogenesis was observed in a study where adult testis tissue was xenografted into immunodeficient mice [107].

#### 5.4.4. Maturation of Testicular Tissue in Culture (Testicular Tissue Organ Culture)

The main benefit of using testicular tissue for starting to develop spermatogenesis in vitro is the presence of testicular somatic cells and the three-dimension (3D) microenvironment suitable for the optimal development of SSCs to complete spermatogenesis. Spermatogenesis development using organ culture to proceed to the meiotic stages was performed in the past without success [108,109,110,111]. In a study performed by Sato et al., they reported a success in inducing the development of fertile sperm using an in vitro culture of small fragments (around 3 mm) of testicular tissue from immature mice [112]. The successful application of the system could in the future be an alternative to autologous SSC transplantation, autologous grafting and xenografting in cases where there is concern about reintroducing malignant cells of the testicular tissue.

#### 5.4.5. Induced Pluripotent Stem Cell

Research is ongoing to induce pluripotent stem cells to differentiate into SSCs. Several groups have now reported that it is possible to produce germ cells from pluripotent embryonic stem cells (ESCs) or induced pluripotent stem cells (iPSCs). Pluripotent stem cells can be obtained through multiple mechanisms. Several often-studied mechanisms include harvesting of embryonal stem cells, reprogramming adult somatic cells to make induced pluripotent stem cells and somatic cell nuclear transfer (SCNT) where a nucleus is inserted into an oocyte. The challenge with the human studies is that it is not possible to test the spermatogenic potential or fertilization potential of putative germ cells, which are the gold standards in animal studies [113,114,115]. Human embryonal stem cells and human pluripotent stem cells have been induced in vitro into haploid cells that resemble spermatid-like cells based on molecular markers, although they have not been functionally assayed [98].

## 6. In Vitro Propagation of SSCs—A Necessary Step before Transplantation

We live in a world of hybrids and therefore another hybrid technique is the in vitro transplantation technique, where donor SSCs are cultured in vitro, then injected into host testis and finally a donor–host mix of tissue fragments induces spermatogenesis in vitro culture. This allows for easy observation of cells compared to pure in vivo xenografting [116]. Oatley et al. transplanted a testicular tissue fragment from a 3 months old human male into castrated mice. The mice were observed after 1 year and it was found that there was some growth of the fragments, spermatogenesis had begun and there were some spermatocytes. This was quicker than the expected speed of development in humans of about 8–10 years of age for spermatocyte development [117].

### 6.1. 2D and 3 Dimensional Culture Systems

The original culture media from Nagano et al. [118] was a standard medium containing Dulbecco Modified Eagle medium (DMEM), fetal bovine serum (FBS), penicillin and streptomycin. The niche may have been partially maintained as donor testis cells were grown together, without isolating SSC specifically. Supplementation of the media has since included addition of GDNF, FGF2, lipid mixtures and co-culture with cells such as testicular stroma [119]. There are several studies in the literature about development of spermatogenesis in vitro in three-dimensional (3D) culture. A study performed by Huleihel et al., was the first to suggest the use of a methylcellulose culture system (MCS) and soft-agar-culture system (SACS) as possible 3D matrices to grow and develop spermatogonia cells in vitro. Using these two novel 3D culture systems (MCS and SACS) it can induce proliferation and differentiation of spermatogonia cells from normal and busulfan-treated immature mice to the meiotic and post-meiotic stages, and even the generation of sperm-like cells [120,121,122]. Same authors demonstrated the presence of spermatogonia cells in testicular biopsies of prepubertal cancer patients before aggressive chemotherapy and their differentiation in MCS to meiotic and post-meiotic cells and, in one case, the generation of sperm-like cells. This suggests 3D culture systems may provide the SSCs with a spatial and microenvironment similar to those present in the seminiferous tubules which are crucial for the development of spermatogenesis such as the presence of testicular somatic cells and the 3D tubular structure. This 3D in vitro culture system still requires optimization in order to efficiently generate sperm [120,121,122,123,124].

### 6.2. 3D Bioprinted Scaffold

To induce the development of spermatogonia cells in order to complete maturation of sperm, a 3D spatial environment is required. This environment must have a similar cellular composition to the seminiferous tubule, and must have a new spatial culture system that is optimized at the different levels of the scaffold and cultured cell. There are several ongoing studies and in 2019 the first report was published of in vitro spermatogenesis in a mouse testicular construct generated by culturing single cell suspensions on 3D bioprinted cell-laden scaffolds and cell-free scaffolds. This new culture system provides alginate-based hydrogel and 3D bioprinting in order to preserve spermatogonia cells in their native 3D spatial and cellular environment to induce complete in vitro spermatogenesis [125].

### 6.3. Testicular Organoids

Th term organoid refers to a 3D structure (up to two millimeters) which is composed of cell aggregates that reorganize after cell dissociation of specific tissue, showing histological and biological activity similar to the original cell. Since there is no vasculature system, the cells’ function depends on having a strong connection to the conditioned media and the nutrients and oxygen provided in vitro [126,127,128]. There are several ongoing studies and one of them refers to a co-culture with Leydig and Sertoli cells in the presence of the extracellular matrix (ECM) from human testis and led to the development of haploid cells after three weeks of culture. Another study showed the development of active human testicular organoids after four weeks of culture (with no similarity to the histology of the testis). An additional study showed the development of mouse spermatogonia cells to meiotic and post meiotic cells in organoids when cultured in fabricated testis-derived scaffolds [129,130,131,132].

Although this technique has been replicated, a study where this organotypic culture was adapted to prepubertal human tissue, with modifications in medium composition, indicated a blockade in maturation of spermatogonia accompanied by a progressive loss of germ cells even though maturation of the somatic cells into a post pubertal phenotype was observed. This observation supports a recent study where major differences were found in the differentiating response of spermatogonia to gonadotropins between monkeys and mice, indicating that functions of genes established to govern spermatogonia differentiation in the mouse may not necessarily translate directly to the primate testis [133].

### 6.4. Microfluid System and Organ-on-Chip Technology

The main limitation of 3D in vitro systems remains the lack of circulatory system. Therefore, organ-on-chip in a microfluid device may overcome this limitation and provide a dynamic condition of nutrients and gas circulation mimicking in vivo conditions.

This system enables the use of small amounts of medium and control over composition, diffusion and temperatures very close to the cells/tissue present in the device. Recently, the development of organ-on-chip of neonate mouse testicular tissue was reported. These systems can lead to success in not only prepubertal cancer patients, but also azoospermic patients where spermatogonia cells are present. The capacity of fertility of the generated sperm in this system still needs to be confirmed, in addition to genetic and epigenetic stability, as is the case with all in-vitro systems [134,135,136].

## 7. Malignant Contamination and In Vitro Spermatogenesis

The risk of reintroducing malignant cells via the graft might be overcome by in vitro spermatogenesis. The consequences of these epigenetic changes on sperm functionality and offspring’s health are unknown. In one study, 20% of patients with leukemia had tumor cells in their testicular tissue prior to the initiation of any gonadotoxic treatment. These results confirm that a testicular biopsy should be done in a cancer patient and could harbor malignant cells [137]. Jahnukainen and colleagues demonstrated that transplantation of as few as 20 leukemic cells was sufficient for disease transmission, leading to terminal leukemia within 3 weeks [138]. This raises serious concerns about cancer relapse in humans and a concern for auto-transplantation of SSCs, especially if these are collected before gonadotoxic treatments. There are several studies in progress which are trying to eliminate acute lymphoid leukemia cells from testis culture [139].

Since infertility is not life threatening and fertility treatments are elective, it is essential that risk of cancer recurrence after transplant be reduced to zero. Fluorescence-activated cell sorting (FACS) and magnetic-activated cell sorting (MACS) strategies to isolate and enrich therapeutic spermatogonia while removing malignant contamination have been explored with mixed results. To date, transplantable human spermatogonia have been recovered in the Ep-CAMlo, THY-1lo, CD49f+, SSEA4+, GPR125+ and CD9+ fractions of human testis cells [140,141,142].

More sensitive PCR-based methods have been described for detection of minimal residual disease (MRD), and this approach has identified malignant contamination in many testicular tissue samples that were preserved for leukemia patients even after negative histology and IHC examination.

The results in the literature are encouraging, but still require caution because some methods were insufficient to remove malignant contamination [143]. It will not be possible to perform comprehensive in vivo testing on patient samples because this would limit the number of samples available for fertility therapy.

In the absence of a definitive and practical test of malignant contamination, alternatives to autologous transplantation are needed for patients with hematogenous cancers, testis cancers or cancers that metastasize to the testes.

## 8. To Omega—Oncogenetic Phenomena

Medicine continues to evolve and become more personalized. The development of a neoplastic cell from a normal progenitor due to structural changes in DNA has been the fundamental basis of the study of the molecular biology of cancer. Research in this field emerged into a new field of cancer genetics called oncogenetics, a branch of genetics describing the cause and effects of genes causing neoplasia. This could prove to be the next frontier of direct personalized medicine [144].

Nowadays, more and more scientists are focused on the genetics of male fertility. Although semen analysis testing is still recognized as a surrogate marker of male fertility, the exponential growth of biomarkers derived from proteomics, epigenomics and genomics has contributed to a new direction of male fertility research. There are several studies in the research literature in which the genetic basis of male fertility after cancer is evaluated, and insights are gained into genetic susceptibility of various cancer therapies, as well as propensity of an individual to regain reproductive function after completing cancer treatment.

There are more than 3000 genes (about 4% of the human genome) expressed in the testicles alone, and hundreds of these genes influence reproductive function in humans [145]. Over 4000 proteins are expressed in the seminal plasma which is why an increased attention has been focused on the proteomes of the testicles, sperm, seminal fluid and epididymis [146]. These proteins might represent a rich source of potential biomarkers for male fertility, and characterization of the reproductive proteome might ultimately lead to significant improvement in the evaluation of the male reproductive tract [147,148,149]. It is been estimated that more than 1000 biomarkers are needed to accurately evaluate male fertility potential. This research can bring existential benefits and lead to a personalized fertility treatment that anticipates reproductive success before and after cancer therapy, a useful tool for clinicians and patients [149].

Research in this field raises a lot of concerns about genetic and epigenetic stability in preservation and culture of testicular tissue. Theoretically, an instability could lead to carcinogenesis and it is mandatory to investigate the epigenetic programming of stem cells in vivo prior to clinical fertilization with any sperm developed from stem cells in vitro [150]. Another field of concern for genetic instability is in vitro culture. SSCs cultured for more than 2 years demonstrate a stability of euploid karyotype with fertile offspring which is considered good stability compared to embryonal stem cells [151]. Other studies have detected genetic changes related to the ageing of SSCs in vitro, where a longer culture time was found to decrease expression of DNA genes which are important to the proper functioning of SSC. These included decreased expression of Bcl6 and Lhx1, which are important for self-renewal, as well as decreased expression of the Thy-1 surface marker. These changes occurred without obvious morphological modifications [151].

The improvement of strategies for the maturation of germ cells in vitro, combined with new molecular techniques for gene editing could be the next scientific keystone for the eradication of genetic diseases such as cancer-related mutations in the offspring of cancer survivors [152,153]. There are several ethic precautions necessary for application of this revolutionary technology. Interventions on the human genome should be admitted only for preventive, diagnostic or therapeutic reasons, therefore avoiding their use in eugenic goals.

Genome editing is a promising tool in preventing disease but there are still many ethical and technical issues of applying this in clinical practice that need to be addressed. If in the future this will prove to be feasible in humans, it could be applied in reproductive medicine to correct disease by causing mutations to avoid their inheritance by offspring [154]. CRISPR/Cas9 genomic edition in gametes from oncological patients subjected to FP may be an interesting approach to avoid the transmission of genetic alterations causing cancer such as BRCA1 / BRCA2 mutated alleles responsible for breast and ovarian cancer. Thus, its application to gametes/gonadal tissue from oncological patients subjected to fertility preservation represents a promising challenge [143,155,156,157,158].

Nevertheless, due to the essential role of gametes to transmit genetic and epigenetic information between generations, assessment of safety and functionality of in vitro generated gametes is mandatory prior to clinical use [159].

## 9. Ethical Issues

Dealing with fertility preservation is an enormous burden as part of cancer treatment and raises a significant number of ethical dilemmas. Our goal in this small chapter is to emphasize the most prominent areas of concern in the context of fertility preservation for pediatric cancer patients and survivors. We searched the literature and found these issues can be divided into ethical concerns at the time of FP treatment and ethical concerns in the future.

Ethical concerns at the time of FP treatment include possible delay of the cancer treatment for the possible future benefit of fertility [160]. Ethical consideration must be given regarding the potential distress and discomfort of the child from the FP treatment, along with surgical and anesthetic risks of the treatments themselves [161]. Consent, assent and serving the child’s best interest are important despite potential parental and provider pressure. The meaning of reproductive health and fertility treatments varies drastically across different cultural and religious backgrounds and involves complicated views on IVF/ICSI, masturbation, involvement of a spermatozoa, uterus donation and the use of tissue in research.

Possible ethical concerns in the future include the impact of FP treatment on future gonadal function, the high cost of treatment and storage of gonadal tissue and insurance coverage. There is also concern regarding offering fair treatment to all patients, as only some may be able to afford this option which gives preference to the wealthy and could lead to potential health disparities [160]. Future ethical issues would also include the possibility of reintroduction of malignant cells with transplantation of gonadal tissue and possible compromised health of the offspring arising from FP treatments [162].

Li et al. reported from 35 articles discussing FP decision-making (11 in the pediatric and adolescent population) that unique ethical issues arise in the pediatric and adolescent population. Considering decision to pursue FP is difficult in the adult population, regret may be greater for parents who are making the decision for their child [163].

There are many areas open for research in this area, including the ethical issues described and the underlying science.

## 10. Conclusions

In our short review we highlighted the importance of fertility for the pediatric male population with cancer, exploring fertility preservation counselling and the improvement of strategies for the maturation of germ cells in vitro, combined with new molecular techniques for gene editing. This could potentially be the next scientific keystone for the eradication of genetic diseases such as cancer- related mutations in the offspring of cancer survivors. The oncofertility field continues to develop worldwide and promising advances for pre and post pubertal male patients with cancer continue to develop.
